# Clinical and Functional Characterization of Novel GALNT3 Mutations in a Chinese Child with Hyperphosphatemic Familial Tumoral Calcinosis

**DOI:** 10.3390/ijms27062767

**Published:** 2026-03-18

**Authors:** Yuan Gao, Cai Zhang, Shimin Wu, Yanqin Ying, Ling Hou, Yan Liang, Xiaoping Luo

**Affiliations:** Department of Pediatrics, Hubei Provincial Key Laboratory of Pediatric Genetic Metabolic and Endocrine Rare Diseases, Hubei Provincial Clinical Research Center for Children’s Growth and Development and Metabolic Diseases, State Key Laboratory for Diagnosis and Treatment of Severe Zoonotic Infectious Disease, Tongji Hospital, Tongji Medical College, Huazhong University of Science and Technology, Wuhan 430030, China; d202382140@hust.edu.cn (Y.G.); linghou@tjh.tjmu.edu.cn (L.H.)

**Keywords:** hyperphosphatemic familial tumoral calcinosis, GALNT3, compound heterozygous mutation, O-glycosylation, protein secretion, phosphate metabolism

## Abstract

Hyperphosphatemic familial tumoral calcinosis (HFTC) is a rare autosomal recessive disorder characterized by hyperphosphatemia and ectopic calcifications. Mutations in GALNT3, which encodes a key enzyme responsible for O-glycosylation of FGF23, represent a major genetic cause of HFTC. This modification is essential for the stability and secretion of FGF23. We investigated a 4-year and 6-month-old Chinese girl with HFTC to characterize the clinical features, identify the causative variants, and explore the underlying pathogenic mechanism. Whole-exome sequencing followed by Sanger validation identified novel compound heterozygous variants in GALNT3 (c.659T>A, p.Ile220Asn and c.1850C>A, p.Ser617*). The patient exhibited hyperphosphatemia with a biochemical profile consistent with FGF23 deficiency, including extremely low intact FGF23 and elevated C-terminal fragments. Functional studies using Western blotting and wheat germ agglutinin affinity chromatography demonstrated that the mutant GALNT3 caused a severe defect in FGF23 O-glycosylation, leading to impaired secretion of intact FGF23. Glycosylated FGF23 was detected only in the medium of cells expressing wild-type GALNT3. These findings indicate that defective O-glycosylation results in failure of FGF23 secretion and functional inactivation. This study expands the mutational spectrum of GALNT3 and provides mechanistic insight into the role of GALNT3 in phosphate homeostasis.

## 1. Introduction

Hyperphosphatemic familial tumoral calcinosis (HFTC) is a rare autosomal recessive disorder characterized by ectopic calcifications, particularly in periarticular soft tissues, and persistent hyperphosphatemia [1]. The disease is caused by mutations in genes critical for phosphate homeostasis, primarily FGF23, GALNT3, and KL [2,3]. Fibroblast growth factor 23 (FGF23), secreted by osteocytes, is a key hormone that promotes renal phosphate excretion and suppresses the synthesis of active vitamin D [4,5]. A crucial step in the production of biologically active, intact FGF23 is its post-translational O-glycosylation, which is mediated by the enzyme UDP-N-acetyl-α-D-galactosamine:polypeptide N-acetylgalactosaminyltransferase 3 (GalNAc-T3), encoded by the GALNT3 gene [6]. This glycosylation modification protects FGF23 from proteolytic cleavage into inactive N-terminal and C-terminal fragments [7]. Consequently, mutations in either FGF23 or GALNT3 disrupt this protective mechanism, leading to reduced levels of intact FGF23, impaired phosphate regulation, and consequent hyperphosphatemia and tissue calcification [8,9].

Clinically, HFTC typically manifests in childhood or adolescence with a triad of symptoms: (1) painful periarticular soft tissue calcifications (tumoral calcinosis), which are often the most disabling feature; (2) dental abnormalities, including short roots and pulp stones; and (3) cutaneous calcifications or ulcerations. Due to its rarity and the inflammatory nature of the calcific masses, HFTC is frequently misdiagnosed as chronic osteomyelitis, rheumatoid arthritis, or other inflammatory conditions, leading to diagnostic delays and inappropriate treatments such as prolonged antibiotic use or unnecessary surgeries. The current management is primarily symptomatic, focusing on a low-phosphate diet and phosphate binders, but these interventions are often incompletely effective, highlighting the need for a deeper molecular understanding to guide targeted therapeutic strategies [1,2,3].

The diagnosis of HFTC remains challenging due to its rarity and the non-specific nature of its initial symptoms, which often mimic other conditions such as osteomyelitis or soft tissue tumors [10,11]. While numerous mutations in FGF23 and GALNT3 have been reported worldwide, the genotypic and phenotypic spectrum, particularly in East Asian populations, is still expanding [12,13,14].

Here, we report a detailed study of a young Chinese girl with HFTC. We describe her diagnostic odyssey from an initial misdiagnosis of osteomyelitis to a definitive genetic diagnosis. Through whole-exome sequencing, we identified novel compound heterozygous mutations in the GALNT3 gene. Furthermore, our functional studies revealed a paradoxical cellular phenomenon: the mutant GALNT3 led to defective O-glycosylation of FGF23, providing novel insights into the pathogenesis of this rare disease.

## 2. Results

### 2.1. Clinical Presentation, Diagnostic Journey, and Family Analysis

**Figure 1:**
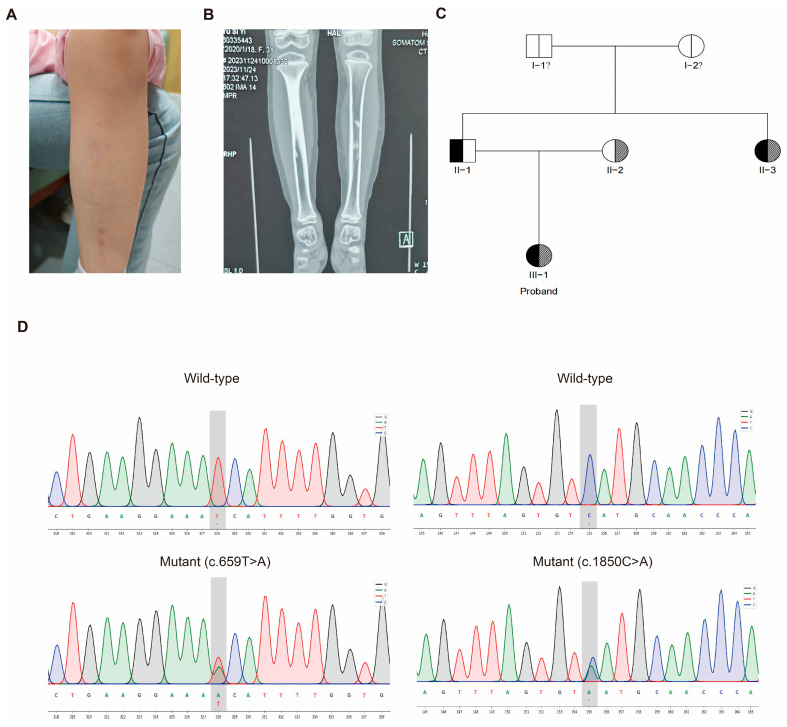
Clinical, genetic, and molecular characterization of the proband with hyperphosphatemic familial tumoral calcinosis (HFTC). (**A**) Clinical photograph of the proband’s left calf, showing swelling and skin changes associated with subcutaneous calcific deposits. (**B**) Radiograph of the lower limbs (anteroposterior view) demonstrating periarticular and soft-tissue calcifications adjacent to the tibia, characteristic of tumoral calcinosis. (**C**) Pedigree of the family. Squares denote males, circles females. Filled symbols represent affected individuals; symbols with a central dot denote heterozygous carriers. The proband (III-1) is a compound heterozygote for two GALNT3mutations. His father (II-1) and mother (II-2) are asymptomatic carriers of the c.659T>A and c.1850C>A mutations, respectively. (**D**) Sanger sequencing chromatograms validating the two GALNT3mutations. Upper panels: Wild-type sequences at nucleotide positions c.659 (**left**) and c.1850 (**right**). Lower panels: Corresponding sequences from the proband, showing the heterozygous c.659T>A (p.Ile220Asn) mutation (**left**) and the heterozygous c.1850C>A (p.Ser617*) mutation (**right**).

The proband was a 4-year and 6-month-old Chinese girl who presented with a history of progressive left calf pain and swelling (Figure 1A). Radiographic examination revealed characteristic periarticular and soft-tissue calcifications, suggestive of tumoral calcinosis (Figure 1B). Biochemical testing confirmed persistent hyperphosphatemia (serum phosphate: 2.08–2.65 mmol/L) and a classic FGF23 dysfunction profile (intact FGF23: 8 pg/mL; C-terminal FGF23: 658 RU/mL), leading to the suspicion of hyperphosphatemic familial tumoral calcinosis (HFTC).

Family history investigation revealed a paternal aunt with adult-onset similar symptoms. Pedigree analysis established an autosomal recessive inheritance pattern (Figure 1C). To identify the genetic etiology, whole-exome sequencing was performed, which identified two novel mutations in the GALNT3gene in the proband: a missense mutation c.659T>A (p.Ile220Asn) and a nonsense mutation c.1850C>A (p.Ser617*). Sanger sequencing confirmed the proband was a compound heterozygote, with each asymptomatic parent carrying one of the mutant alleles (Figure 1D), thus providing the molecular confirmation for the diagnosis of HFTC. The detailed clinical course and laboratory findings are summarized in Table S1.

### 2.2. Structural and Functional Analysis of the Novel GALNT3 p.Ile220Asn Variant

To investigate the structural and functional impact of the novel p.Ile220Asn missense mutation, we performed a two-tiered computational analysis.

**Figure 2:**
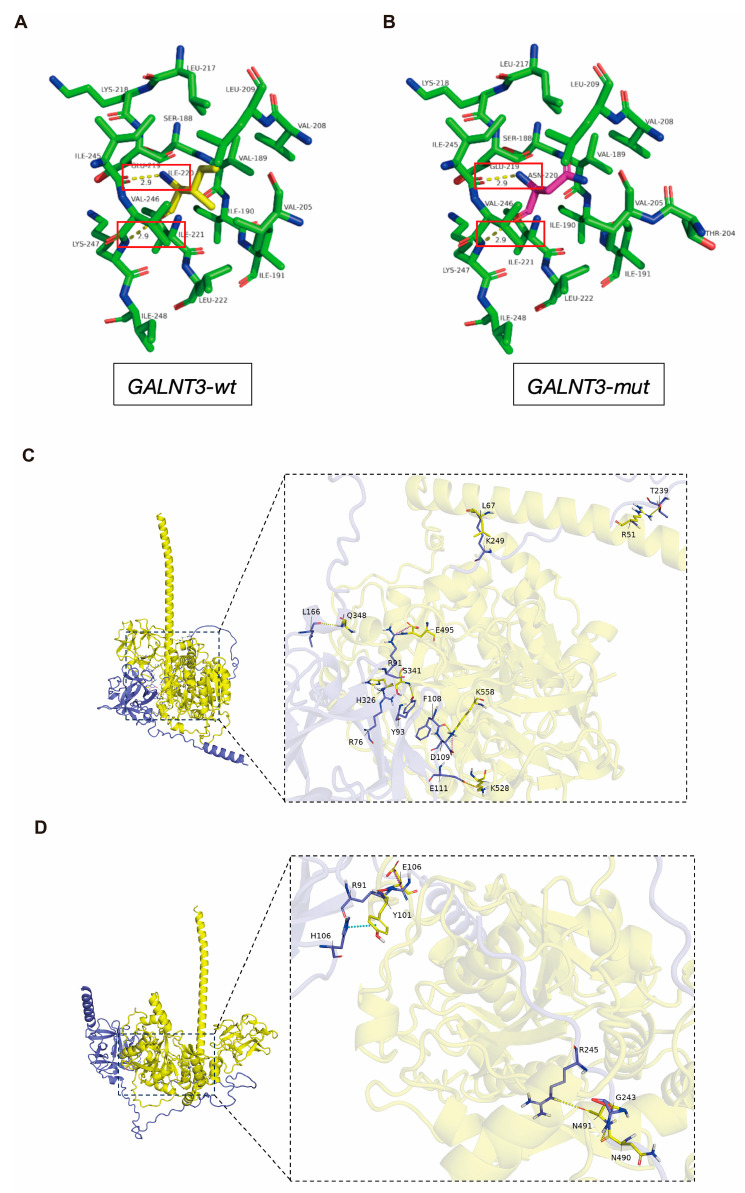
Structural comparison of wild-type and mutant GALNT3 protein. (**A**) Three-dimensional structure of the glycosyltransferase domain of wild-type GALNT3 (GALNT3-wt), with key interacting residues highlighted in red boxes and colored by atom type (carbon: green, nitrogen: blue, oxygen: red). (**B**) Corresponding structure of the mutant GALNT3 harboring the p.Ile220Asn substitution (GALNT3-mut), with the same color scheme and red boxes indicating critical interaction regions. (**C**) Overall interface between the GALNT3 glycosyltransferase domain (yellow) and the FGF23 substrate peptide (blue). The zoomed-in panel highlights key residues involved in substrate recognition and binding. (**D**) Interface for the mutant GALNT3 (p.Ile220Asn) with FGF23. The mutated residue (Asn-220, yellow) is shown forming new interactions with surrounding residues, potentially altering substrate positioning and catalytic efficiency.

We first compared the three-dimensional structures of the wild-type (WT) and mutant (I220N) GALNT3 glycosyltransferase domains under resting conditions. The analysis revealed that the substitution of a hydrophobic isoleucine with a polar asparagine at residue 220 did not alter the local hydrogen-bonding network or the global protein fold (Figure 2A,B). Key interatomic distances were preserved. Therefore, the p.Ile220Asn substitution is predicted not to cause a major disruption to the overall protein structure or stability.

Precisely because the global structure appeared intact, we used protein–protein docking to specifically assess the mutation’s effect on substrate (FGF23) recognition, a key functional parameter. Docking simulations between the GALNT3 catalytic domain and full-length FGF23 were performed. The results indicated that the I220N mutation reduced the predicted binding affinity for FGF23 (Figure 2C,D). Detailed analysis of the docking poses suggested that the mutation altered the interface geometry and intermolecular interactions, potentially compromising the precise positioning of the FGF23 substrate for efficient glycosylation. This computational finding provides a structural rationale for a defect in enzyme–substrate recognition, aligning with the functional deficiency observed in our in vitro assays.

### 2.3. Plasma FGF23 Quantification and In Vitro Functional Analysis Reveal a Paradoxical Secretion Increase with Loss of Function

**Figure 3:**
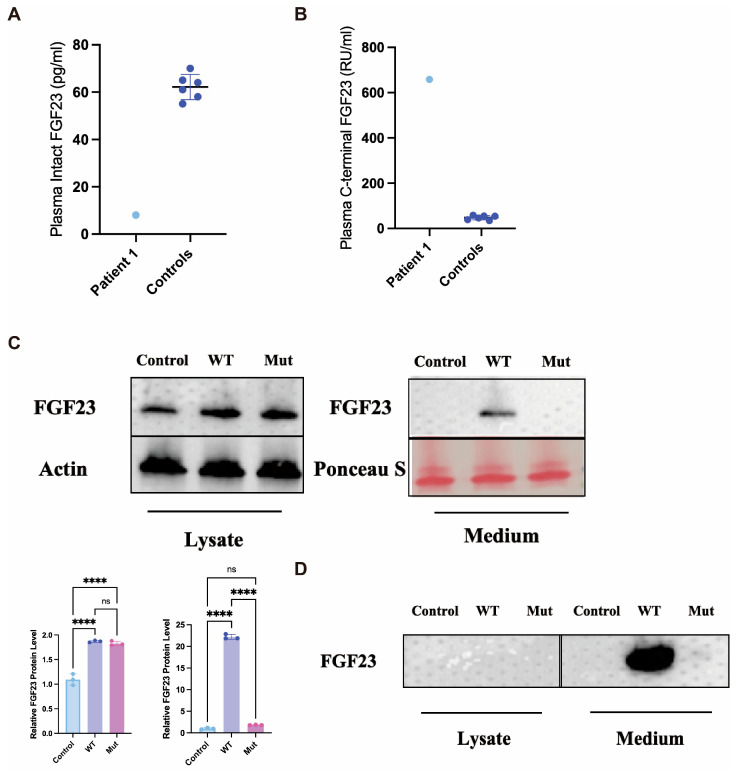
Biochemical profile and functional analysis of FGF23 in the proband. (**A**) Plasma levels of intact FGF23 in the proband (Patient 1) and age-matched healthy controls. (**B**) Plasma levels of C-terminal FGF23 fragment in the proband and controls. (**C**) In vitro analysis of FGF23 expression, secretion, and glycosylation status. (Upper panels) Western blot analysis of cell lysates (Left panel) and conditioned media (Right panel) from cells expressing wild-type (WT) or mutant (Mut) GALNT3, or a control vector (Control). β-Actin serves as a loading control for cell lysates. Ponceau S staining of the membrane is shown as a loading control for the concentrated medium samples. (Lower panels) Quantification of FGF23 protein levels in lysate and medium samples, normalized to their respective loading controls and expressed relative to the WT group (set to 1.00). Data represent mean ± SEM (*n* = 3 independent experiments). Statistical significance was assessed by one-way ANOVA followed by Tukey’s post hoc test: **** *p* < 0.0001 vs. WT in the same compartment (lysate or medium); ns, not significant. (**D**) Western blot analysis of FGF23 in conditioned media following WGA affinity chromatography to assess glycosylation status.

To delineate the pathogenic mechanism of the GALNT3 mutations, we first assessed the circulating FGF23 profile in the proband. Consistent with the loss-of-function nature of HFTC, the plasma level of biologically active intact FGF23 was markedly reduced (8 pg/mL) compared to age-matched healthy controls (62.2 ± 5.3 pg/mL, *n* = 6) (Figure 3A). Conversely, the level of the inactive C-terminal FGF23 fragment was profoundly elevated (658 RU/mL vs. 47.8 ± 8.3 RU/mL in controls) (Figure 3B), indicating enhanced proteolytic cleavage of the hormone.

To elucidate the cellular basis of this biochemical profile, we conducted in vitro functional studies. Western blot analysis of both cell lysates and conditioned media from hFOB1.19 cells transfected with wild-type (WT) or mutant (N162D) constructs was performed. The expression levels of WT and mutant FGF23 within the cell lysates were comparable. However, crucially, secreted intact FGF23 was detected only in the conditioned media from cells expressing the WT protein, and was absent in the media from cells expressing the mutant FGF23 (Figure 3C,D). This indicates that the N162D mutation impairs the secretion of intact FGF23. To determine if this secretion defect was linked to impaired O-glycosylation, we performed wheat germ agglutinin (WGA) affinity chromatography. The analysis of the O-glycosylated protein fractions revealed that glycosylated, intact FGF23 was exclusively detectable in the media from WT-expressing cells, and was undetectable in both the cell lysates and media from mutant-expressing cells. These combined findings demonstrate that the N162D mutation prevents FGF23 O-glycosylation, which in turn leads to a failure in the secretion of the stable, intact hormone.

This combination of findings—a glycosylation defect leading to impaired secretion of FGF23 in vitro, coupled with low intact FGF23 and high C-terminal fragments in patient plasma—provides a coherent pathogenic mechanism. It demonstrates that the disease is caused by a qualitative defect in post-translational modification, which results in a failure to secrete the stable, functional hormone. The non-glycosylated FGF23, unable to be secreted efficiently, is presumably retained or degraded intracellularly. Any minimal amount that might reach the circulation would be intrinsically unstable and highly susceptible to proteolysis, ultimately leading to the characteristic loss-of-function biochemical phenotype and consequent hyperphosphatemia.

## 3. Discussion

**Figure 4:**
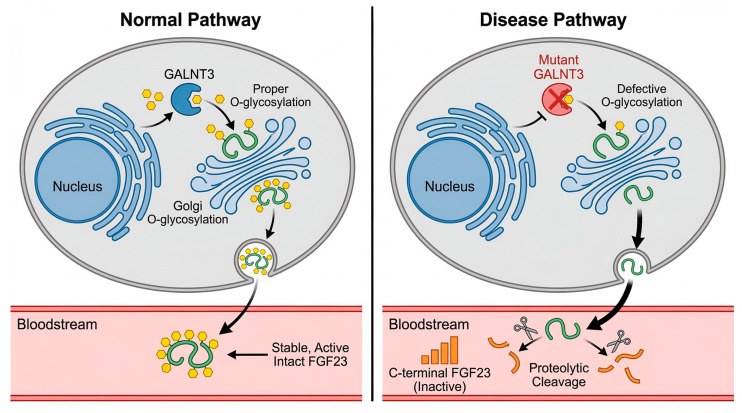
Molecular Mechanism of FGF23 Dysfunction in GALNT3-Related Hypophosphatemic Rickets with Hypercalciuria.

In this study, we present a comprehensive clinical, genetic, and functional analysis of a pediatric HFTC case caused by novel compound heterozygous mutations in the GALNT3 gene (c.659T>A, p.Ile220Asn and c.1850C>A, p.Ser617*). Our findings not only confirm the indispensable role of GALNT3-mediated glycosylation for FGF23 function but also delineate the pathogenic mechanism by demonstrating that the p.Ile220Asn mutation disrupts O-glycosylation, thereby preventing the secretion of stable, functional FGF23 protein (Figure 4).

The clinical presentation of our patient, characterized by early-onset bone pain, hyperphosphatemia, and ectopic calcifications, is consistent with classic HFTC [2,15]. However, her initial misdiagnosis as osteomyelitis underscores a significant clinical challenge. The inflammatory features and bone lesions seen in HFTC can closely mimic infectious or autoimmune osteomyelitis [10,11,16]. Our case highlights the importance of considering metabolic bone disorders, particularly in the presence of persistent hyperphosphatemia, to avoid diagnostic delays and inappropriate treatments such as prolonged antibiotic therapy or surgical intervention.

Our case also underscores the importance of precise diagnosis and genetic counseling in East Asian populations. The compound heterozygous mutations (c.659T>A, p.Ile220Asn and c.1850C>A, p.Ser617*) identified in this study further expand the spectrum of GALNT3 mutations in East Asians [12,13]. Although HFTC is rare globally, its specific genotype–phenotype correlations in East Asian populations may possess distinct characteristics. Enhancing clinician awareness of this disorder in East Asia is crucial to prevent misdiagnoses, such as osteomyelitis, as encountered in our case. Furthermore, a clear molecular diagnosis enables accurate genetic counseling for the family, informs recurrence risks, and facilitates screening of symptomatic relatives, paving the way for early intervention and management.

The core mechanistic insight from our work stems from the in vitro functional characterization of the p.Ile220Asn missense mutation. Our data reveal a clear pathogenic sequence: first, the mutation completely abrogates the O-glycosylation of FGF23, as definitively shown by its failure to bind to WGA lectin. Second, and critically, this glycosylation defect directly impairs the cellular secretion of the stable, intact FGF23 protein. While the mutant FGF23 was expressed intracellularly at levels comparable to the wild-type, the glycosylated, intact form was exclusively detected in the conditioned media of cells expressing the wild-type protein, and was absent from the media of mutant-expressing cells. This finding provides a direct cellular explanation for the patient’s plasma profile. The non-glycosylated FGF23, failing to be secreted efficiently, is presumably retained and/or degraded intracellularly. Any minimal amount that might reach the circulation would, as supported by prior literature [7,17,18], be exquisitely susceptible to proteolytic cleavage, thereby generating the observed excess of C-terminal fragments and the deficiency of active, intact hormone. Thus, our study demonstrates that the disease mechanism is a “qualitative” defect in post-translational modification that leads to a “quantitative” failure in hormone secretion, moving beyond genotype–phenotype correlation to a defined cellular pathophysiology. While our data strongly support this primary hypothesis of a defect in GALNT3’s catalytic function, we acknowledge that our study does not fully delineate all potential downstream cellular consequences, such as whether the mutation also alters intracellular trafficking kinetics. Future studies involving direct enzymatic assays, structural analyses, and in vivo models will be valuable to precisely map the affected molecular steps and explore alternative pathways.

Structurally, the p.Ile220Asn mutation is located in the glycosyltransferase domain. Our modeling suggested it does not drastically alter the protein’s fold, implying that the defect is likely in the catalytic activity or substrate recognition of the enzyme, a hypothesis supported by our functional data. The second mutation, c.1850C>A (p.Ser617*), is a nonsense mutation expected to lead to a truncated protein and loss of function, following the classical null mechanism.

This study is distinguished from prior reports by three key aspects: (1) the identification of novel compound heterozygous GALNT3 mutations (c.659T>A, p.Ile220Asn and c.1850C>A, p.Ser617) in an East Asian population; (2) the functional demonstration of a “qualitative defect” mechanism, where mutant GALNT3 causes increased secretion of a hypoglycosylated, dysfunctional FGF23; and (3) the integration of a diagnostic odyssey with genetic and mechanistic analysis to provide a comprehensive model for rare disease investigation.

A limitation of our study is its focus on a single proband. While we provide a deep mechanistic analysis, the findings await confirmation in larger cohorts. Furthermore, the precise reason for the observed increase in FGF23 secretion in the presence of the mutant GALNT3 remains to be fully elucidated and represents an interesting direction for future research.

Strengths of this study include its integrated approach (clinical, genetic, and functional) and the elucidation of a novel pathogenic mechanism—enhanced secretion of a hypoglycosylated FGF23—that directly supports the “qualitative defect” hypothesis in HFTC. Future perspectives should focus on validating the predicted substrate-binding defect through structural studies, exploring the cause of increased secretion, and collecting multi-ethnic genotype–phenotype data to improve diagnosis and therapy.

In conclusion, we identified novel compound heterozygous GALNT3 mutations in a Chinese child with HFTC. Our study expands the genetic spectrum of HFTC and, more importantly, elucidates a pathogenic mechanism where mutant GALNT3 causes a qualitative defect in FGF23, characterized by enhanced secretion of a hypoglycosylated, unstable protein. This work reinforces the indispensability of GALNT3-mediated glycosylation for FGF23 function and provides valuable insights for the diagnosis and genetic counseling of families affected by this rare disorder.

## 4. Methods

### 4.1. Subjects and Ethical Compliance

This study involved a Chinese family with hyperphosphatemic familial tumoral calcinosis (HFTC). The proband was a 4-year and 6-month-old girl. Clinical and biochemical data were collected from the proband and her family members.

The study protocol was approved by the Ethics Committee of Tongji Hospital, Tongji Medical College, Huazhong University of Science and Technology (TJ-IRB20210871, approved on 23 August 2021). Written informed consent was obtained from the parents or legal guardians. All procedures conformed to the principles outlined in the Declaration of Helsinki.

### 4.2. Genetic Analysis

Genomic DNA was extracted from peripheral blood samples using a HiPure Tissue & Blood DNA Kit (Magen, Guangzhou, China). Whole-exome sequencing was performed on the proband. Candidate variants were filtered and prioritized based on the clinical phenotype. The identified mutations in the GALNT3 gene were subsequently validated and confirmed in the proband and her parents by Sanger sequencing using an automated sequencer (ABI 3730XL; Applied Biosystems, Foster City, CA, USA).

Whole-exome sequencing (WES) was performed on the proband. Briefly, a sequencing library was prepared using the KAPA HyperExome Library Preparation Kit (Roche, Basel, Switzerland) following the manufacturer’s protocol. The enriched exome libraries were sequenced on an Illumina NovaSeq 6000 platform (Illumina, San Diego, CA, USA), generating 150 bp paired-end reads. The average sequencing depth across the target regions was >100×, with >95% of the target bases covered at ≥20×. Bioinformatic analysis was conducted as follows: raw sequencing reads were aligned to the human reference genome (GRCh38/hg38) using BWA-MEM (v0.7.17). Variant calling was performed with the GATK (v4.2) Best Practices pipeline. Identified variants were annotated and filtered against public databases (gnomAD and 1000 Genomes) and in-house control databases. Variants were prioritized based on their frequency (<1% in population databases), predicted pathogenicity, and relevance to the clinical phenotype (autosomal recessive inheritance, hyperphosphatemia, ectopic calcification).

The candidate compound’s heterozygous mutations in the GALNT3 gene (c.659T>A and c.1850C>A) were subsequently validated and confirmed in the proband and her parents by Sanger sequencing. PCR amplification was performed using 2× Taq Master Mix (Vazyme, Nanjing, China), and the products were purified and sequenced bidirectionally on an ABI 3730XL automated sequencer (Applied Biosystems, Foster City, CA, USA). The obtained chromatograms were analyzed and compared to the reference sequence (NM_004482.4) using SnapGene software (v6.0.2).

The functional impact of the identified variants was predicted using in silico tools, including MutationTaster (http://www.mutationtaster.org/) and PolyPhen-2 (http://genetics.bwh.harvard.edu/pph2/) (accessed on 18 August 2025). The variants were checked against the Human Gene Mutation Database (HGMD) and ClinVar to confirm their novelty.

### 4.3. Plasmid Construction and Cell Culture

The wild-type (WT) human GALNT3 cDNA was cloned into a mammalian expression vector. The c.659T>A (p.Ile220Asn) mutation was introduced into the WT GALNT3 plasmid using a site-directed mutagenesis kit (TransGen Biotech, Beijing, China). The sequences of all constructs were verified by Sanger sequencing.

The human fetal osteoblast cell line hFOB1.19 (CRL-11372; American Type Culture Collection, ATCC, Manassas, VA, USA) was cultured in Dulbecco’s Modified Eagle Medium/Nutrient Mixture F-12 (DMEM/F-12) (Cat. No. 11330032; Gibco, Waltham, MA, USA) supplemented with 10% fetal bovine serum (FBS) (Cat. No. 10099141, South America Origin; Gibco, USA) and 1% penicillin/streptomycin (Cat. No. 15140122; Gibco, USA). Cells were maintained in a humidified incubator with 5% CO_2_ at 37 °C. All experiments were performed with cells at passages between 5 and 15 to ensure phenotypic stability. Prior to functional assays, cells were seeded in appropriate culture vessels and grown to 80–90% confluence.

### 4.4. Protein Quantification and Sample Preparation

Total protein concentration of cell lysates and conditioned media was determined using the Pierce™ BCA Protein Assay Kit (Cat. No. 23225; Thermo Fisher Scientific, Waltham, MA, USA) according to the manufacturer’s instructions, with absorbance measured at 562 nm using a SpectraMax iD3 Multi-Mode Microplate Reader (Molecular Devices, San Jose, CA, USA). Samples were prepared for electrophoresis by mixing with 4× Laemmli Sample Buffer (Bio-Rad, Hercules, CA, USA) containing 10% β-mercaptoethanol (Sigma-Aldrich, St. Louis, MO, USA) and denatured at 95 °C for 5 min.

### 4.5. Western Blot Analysis

Equal amounts of protein (20 µg per lane) were separated by SDS-PAGE on 4–20% Mini-PROTEAN^®^ TGX™ Precast Protein Gels (Cat. No. 4561094; Bio-Rad, USA) using 1× Tris/Glycine/SDS Running Buffer (Bio-Rad, USA). Proteins were then electrophoretically transferred to 0.45 µm PVDF membranes (Cat. No. 1620177; Bio-Rad, USA) using the Trans-Blot^®^ Turbo™ Transfer System (Bio-Rad, USA) with a mixed molecular weight program (25 V, 1.0 A, 30 min). Membranes were blocked for 1 h at room temperature with 5% (*w*/*v*) non-fat dry milk (Bio-Rad, USA) in Tris-buffered saline containing 0.1% Tween-20 (TBST). Subsequently, membranes were incubated overnight at 4 °C with the following primary antibodies (diluted in blocking buffer): rabbit anti-FGF23 (1:1000; Cat. No. ab200123, Abcam, Cambridge, UK) and mouse anti-β-Actin (1:5000; Cat. No. 66009-1-Ig, Proteintech, Rosemont, IL, USA). After three 10 min washes with TBST, membranes were incubated for 1 h at room temperature with the corresponding HRP-conjugated secondary antibodies: goat anti-rabbit IgG (1:5000; Cat. No. 7074S, Cell Signaling Technology, Danvers, MA, USA) or horse anti-mouse IgG (1:5000; Cat. No. 7076S, Cell Signaling Technology, USA). Protein bands were visualized using the SuperSignal™ West Pico PLUS Chemiluminescent Substrate (Cat. No. 34580; Thermo Fisher Scientific, USA) and imaged with a ChemiDoc™ MP Imaging System (Bio-Rad, USA). Densitometric analysis of band intensities was performed using ImageJ software (v1.53k, National Institutes of Health, Bethesda, MD, USA).

### 4.6. Wheat Germ Agglutinin (WGA) Affinity Chromatography

To assess O-glycosylation, Wheat Germ Agglutinin (WGA) Agarose (Cat. No. AL-1023; Vector Laboratories, Burlingame, CA, USA) was used. Conditioned media samples (containing 200 µg total protein) were diluted in 1× TBS (pH 7.4) and incubated with 50 µL of pre-equilibrated WGA-agarose beads for 2 h at 4 °C with end-over-end rotation. The beads were then washed three times with 1× TBS containing 0.1% Tween-20. Bound glycoproteins were eluted by boiling the beads in 1× Laemmli Sample Buffer for 5 min. Both the flow-through (unbound) and eluted (bound) fractions, along with the original input, were analyzed by Western blot as described above.

### 4.7. Structural and Computational Modeling

To assess the functional impact of the p.Ile220Asn (I220N) mutation, protein–protein docking was performed. The three-dimensional structures of the wild-type (WT) and I220N mutant forms of the human GALNT3 glycosyltransferase domain (UniProt ID: Q14435) were obtained from the AlphaFold database (AF-Q14435-F1). The I220N mutation was introduced in silico using PyMOL (version 2.5.x). The structure of the full-length human FGF23 (UniProt ID: Q9GZV9) was also retrieved from AlphaFold (AF-Q9GZV9-F1). Protein–protein docking between (1) WT GALNT3 and FGF23, and (2) mutant (I220N) GALNT3 and FGF23, was performed using the HDOCK server (http://hdock.phys.hust.edu.cn/). Default parameters were used. The resulting docking poses were analyzed, and the predicted binding affinity (in arbitrary HDOCK score units) for the top-ranking complex was compared between the WT and mutant systems to evaluate the effect of the mutation on the protein–protein interaction.

### 4.8. Statistical Analysis

Densitometric analysis of Western blot bands was performed using ImageJ software (National Institutes of Health, USA). Data from at least three independent experiments are presented. Statistical significance was determined using Student’s t-test, with a *p*-value < 0.05 considered significant.

## Data Availability

All data generated or analyzed during this study are included in this published article.

## Supplementary Materials

The following supporting information can be downloaded at: https://www.mdpi.com/article/10.3390/ijms27062767/s1.
