# Supplementary material for: Clinical and Functional Characterization of Novel GALNT3 Mutations in a Chinese Child with Hyperphosphatemic Familial Tumoral Calcinosis
Source: Int J Mol Sci. 2026 Mar 18;27(6):2767. doi: 10.3390/ijms27062767 (PMC13026893; doi:10.3390/ijms27062767)
Supplement: Supplementary file 1 [file ijms-27-02767-s001.zip › ijms-4173461-supplementary.pdf]

Table S1. Longitudinal Clinical, Biochemical, and Genetic Profile of the Proband with Hyperphosphatemic Familial Tumoral Calcinosis (HFTC).

Category	Details (Patient 1, Female; Date of Birth: Jan 18, 2020)	Interpretation / Clinical Significance
**Initial Presentation (Nov 2023, Age 3y10m)**	Chief Complaint: Left calf pain, swelling, warmth, and limping for 6 days. No fever or trauma.<br>Initial Diagnosis: Left tibial osteomyelitis.<br>Imaging (PET-CT, Dec 2023): Bilateral femoral and tibial medullary cavities showed patchy high-density shadows, with slightly increased glucose metabolism in the left tibia (SUVmax=3.0).	Presentation mimicked acute osteomyelitis. PET-CT revealed multifocal bone involvement and increased metabolism, supporting an inflammatory/infectious hypothesis.
**Diagnostic Misadventure & Work-up**	Histopathology (Bone biopsy, Dec 2023): Reactive bone hyperplasia with inflammatory cell infiltration, interpreted as chronic osteomyelitis.<br>Treatment Received: Underwent left tibial fenestration and drainage (Dec 15, 2023).<br>Early Biochemical Markers (2023-2024): ESR fluctuated (up to 60 mm/h). Blood/wound cultures were negative.	Extensive work-up initially supported an inflammatory etiology. Surgical intervention was performed based on the misdiagnosis.
**Key Biochemical Hallmarks (Longitudinal, 2024-2025)**	1. Serum Phosphate (PHOS): Persistently elevated above the pediatric reference range (1.45-2.10 mmol/L). Values ranged from 2.08 to 2.65 mmol/L across multiple tests.<br>2. Serum Calcium (Ca): Consistently within the normal range (2.23-2.80 mmol/L).	Persistent hyperphosphatemia with normocalcemia is the biochemical hallmark. The classic FGF23 imbalance (low intact, high C-terminal) is pathognomonic for HFTC and confirmed the diagnosis.

	3. FGF23 Profile (Definitive Test): Intact FGF23: 8 pg/mL (Extremely low); C-terminal FGF23: 658 RU/mL (Extremely high).<br>4. Other Markers: Renal/liver function normal. Bone turnover markers (e.g., ALP) were variably elevated.	
**Genetic Diagnosis (Established Jun 2024)**	Whole-exome sequencing revealed compound heterozygous mutations in the GALNT3 gene:<br>1. c.659T>A (p.Ile220Asn) — a novel missense variant.<br>2. c.1850C>A (p.Ser617*) — a nonsense variant.<br>Parental Testing: Both parents were confirmed as heterozygous carriers.	Definitive molecular diagnosis established. The mutations explain the FGF23 glycosylation defect and confirm autosomal recessive inheritance.
**Post-Diagnosis Disease Course & Management (2024-2025)**	1. Diagnosis Evolution: Clinical diagnosis updated from “osteomyelitis/CRMO” to “Hyperphosphatemic Familial Tumoral Calcinosis (HFTC)”.<br>2. Treatment Shift: From surgical/intervention to medical management:<br>• Immunomodulation: Prednisone, Methotrexate, Tofacitinib.<br>• Dietary: Phosphate-restricted diet (formal nutrition consultation).<br>• Supportive: Analgesics, Vitamin D, Folic acid.	Illustrates the diagnostic odyssey and shift to disease-specific management. The persistent hyperphosphatemia underscores the limited efficacy of current therapies in correcting the underlying metabolic defect.

	3. Follow-up (2025): Patient reported occasional calf pain without affecting ambulation. Latest serum phosphate (Jun 2025) was 2.18 mmol/L (still elevated).	
**Family History**	Paternal Aunt: Adult-onset case with lower limb symptoms, clinically diagnosed with "tumoral calcinosis" and treated with a similar immunomodulatory regimen (Prednisone, Methotrexate, Tofacitinib).	Supports autosomal recessive inheritance and suggests variable age of onset within the family.
